# Study on Microstructure and Wear Resistance of Multi-Layer Laser Cladding Fe901 Coating on 65 Mn Steel

**DOI:** 10.3390/ma18153505

**Published:** 2025-07-26

**Authors:** Yuzhen Yu, Weikang Ding, Xi Wang, Donglu Mo, Fan Chen

**Affiliations:** 1 School of Mechanical Engineering, Yancheng Institute of Technology, Yancheng 224051, China; dingweikang2023@163.com (W.D.); wangxi2020@ycit.edu.cn (X.W.); 17805240737@163.com (F.C.); 2 School of Mechanical and Power Engineering, Nanjing Tech University, No. 30 South Puzhu Road, Nanjing 211800, China; modonglu@126.com

**Keywords:** laser cladding, Fe901 alloy coatings, microstructure, microhardness, wear resistance

## Abstract

65 Mn is a high-quality carbon structural steel that exhibits excellent mechanical properties and machinability. It finds broad applications in machinery manufacturing, agricultural tools, and mining equipment, and is commonly used for producing mechanical parts, springs, and cutting tools. Fe901 is an iron-based alloy that exhibits excellent hardness, structural stability, and wear resistance. It is widely used in surface engineering applications, especially laser cladding, due to its ability to form dense and crack-free metallurgical coatings. To enhance the surface hardness and wear resistance of 65 Mn steel, this study employs a laser melting process to deposit a multi-layer Fe901 alloy coating. The phase composition, microstructure, microhardness, and wear resistance of the coatings are investigated using X-ray diffraction (XRD), optical microscopy, scanning electron microscopy (SEM), Vickers hardness testing, and friction-wear testing. The results show that the coatings are dense and uniform, without visible defects. The main phases in the coating include solid solution, carbides, and α-phase. The microstructure comprises dendritic, columnar, and equiaxed crystals. The microhardness of the cladding layer increases significantly, with the multilayer coating reaching 3.59 times the hardness of the 65 Mn substrate. The coatings exhibit stable and relatively low friction coefficients ranging from 0.38 to 0.58. Under identical testing conditions, the wear resistance of the coating surpasses that of the substrate, and the multilayer coating shows better wear performance than the single-layer one.

## 1. Introduction

The soil-contacting parts of agricultural machinery are frequently exposed to soil and other abrasive particles without lubrication, creating a harsh working environment [1]. Abrasive wear caused by soil contact is a primary contributor to agricultural machinery obsolescence [2]. 65 Mn is a high-quality carbon structural steel with significant application value in machinery manufacturing due to its excellent mechanical properties and processing performance, agricultural machinery, mining equipment, etc., and is commonly used in the manufacture of mechanical parts, springs, cutting tools, etc. [3,4]. One of the primary causes of failure in soil-touching components of agricultural machinery is wear and tear. During operation, rotary tillage knives are exposed to abrasive impacts from soil, sand, gravel, and weeds. This constant exposure to abrasion can cause the body of the knives to fail, resulting in reduced plowing efficiency and increased plowing costs [5]. To address this issue, it is important to consider materials and designs that can withstand these abrasive forces. The study of surface wear treatment of soil-touching parts of agricultural machines has gained attention due to the advancement of agricultural mechanization. Therefore, improving the wear resistance of the surface of rotary tillage knives is of great significance in enhancing the productivity of agricultural mechanization. Laser cladding technology can be utilized to create a high-performance alloy layer on the surface of inexpensive metal materials, thereby enhancing their wear and corrosion resistance. When it comes to improving the surface properties of metals, the process of laser deposition of various types and forms of coatings is superior in terms of both performance and cost compared to the various spray methods available on the market. Currently, laser technology is also being developed to improve the hardness and wear resistance of agricultural machinery. It is also used to clad components that come into contact with the soil. This is a new application field for laser cladding technology [6].

Multi-layer laser cladding technology can form thick coatings on the surface of the substrate by layer-by-layer deposition, which provides an effective solution for the preparation of wear-resistant and high hardness coatings [7,8]. Multi-layer laser cladding can produce materials with a uniform composition and low levels of segregation, which helps to avoid the detrimental effects of elemental segregation on the material’s resistance to wear and oxidation at high temperatures associated with conventional manufacturing techniques [9]. Scholars have extensively studied the performance of multi-layer laser melt coatings, which are commonly used for repairing rollers and pistons. In their study, Bykovskiy et al. [10] analyzed the constituents and distribution of hardness in double-layer coatings with various orientations of the second layer trajectory. In their study, Xu et al. [11] examined the impact of multilayer fusion cladding methods on crack susceptibility. They investigated the laser multilayer fusion cladding process using constant and gradient chemical compositions. The crack sensitivity is lower in materials with a gradient distribution of WC (Tungsten Carbide) particles compared to those with a constant distribution of chemical composition.

Iron-based coatings are commonly used in industrial processing and manufacturing due to their superior hardness, improved wear properties, and lower cost [12]. Mixing other metal factors or reinforcing granules in iron-based metal systems can effectively enhance the performance of iron-based alloy coatings. Ramiro et al. [13] demonstrated that defect-free iron-based coatings can be generated using laser cladding without heating the substrate. In their study, Zhao et al. [14] created three distinct iron-based coatings using pure nickel powder, nickel-based alloy powder, and other iron-based alloy powders. They discovered that the iron-based coatings that incorporated pure nickel powder exhibited defects such as pore cracks. In addition, they observed that the incorporation of nickel-based powder alloy powder reduced the cracking tendency of the coating. Chen et al. [15] demonstrated that the hardness of an iron-based cladding coating on a pure titanium substrate could reach up to 800 HV which is 4–5 times higher than that of the substrate. This increase in hardness can be attributed to the formation of hard particles, such as Fe_2_Ti, Fe_2_B, Fe_3_Si, and Ti_2_Ni. Ding et al. [16] analyzed the friction mechanism and wear properties of wheel-rail materials. They found that the addition of lanthanum oxide to the laser-melted iron-based coatings could refine the matrix structure of the coatings, which improved the performance and durability of the wheel-rail materials. Zhang et al. [17] synthesized the V8C7 strengthened phase in situ in an iron-based coating to produce a defect-free coating with higher microhardness and wear resistance. Lin et al. [18] carefully investigated the effect of titanium on the iron-based coatings produced by comparing the microstructure and morphology of iron-based coatings containing titanium with different contents. The presence of titanium reduces the dendritic organization and increases the quantity of equiaxed crystals, thus changing the microstructure. However, the reduction in size causes a decrease in hardness and an increase in the crystalline phase. Wang et al. [19] demonstrated that the inclusion of Mo in laser-melted iron-based composite coatings can impact the growth characteristics of carbides. The addition of a certain mass of Mo results in smaller carbide size in the generated coating, but a larger number of carbides during the laser melting process. This, in turn, enhances the crack resistance of the iron-based composite coatings. Xiao et al. [20] state that the formation of austenite in iron-based alloy coatings varies depending on the nickel content, which affects the growth of dendrites containing higher amounts of chromium. As the nickel content increases, the microhardness of the alloy decreases, but the electrochemical corrosion resistance increases. Lu et al. [21] proposed a three-laser scanning approach to achieve iron-based coatings that are free of cracks through laser melting.

In conclusion, it is feasible to improve the surface properties of 65 Mn steel by laser cladding with iron-based coatings. Based on actual usage conditions and requirements, iron-based coatings are prepared and their properties are thoroughly analyzed. In this study, considering the basic properties of the elements added to the Fe901 alloy powder, a reciprocal deposition strategy, specifically multi-layer laser cladding, was employed. We used Fe901 alloy powder to prepare a hard, wear-resistant coating on the surface of 65 Mn steel. During the rapid melting and solidification process, the coating alternated between hardness and softness, overcoming the drawbacks of high cost and poor flow associated with traditional coating manufacturing methods. Comparative tests showed that the wear rate of the multi-layer laser cladding coating is lower than that of the 65 Mn substrate, significantly improving the wear resistance and service life of the 65 Mn steel.

## 2. Experimental Procedures

### 2.1. Preparation of Materials and Samples

The experiment utilized 65 Mn steel as the substrate material. Table 1 shows the chemical composition of 65 Mn substrate. The steel was isothermally quenched at 850 °C for 10 min, followed by tempering at 200 °C according to the nitrate and nitrite ratio. In a study by Daniel Fabijanic et al. [22], it was discovered that the coating’s plasticity and toughness were negatively affected by the presence of non-tempered martensite between the HAZ (Heat-Affected Zone) and the fused cladding layer. Therefore, the samples were tempered after fusion cladding in this experiment. The dimensions of the specimen were 20 mm × 20 mm × 5 mm.

**Figure 1:**
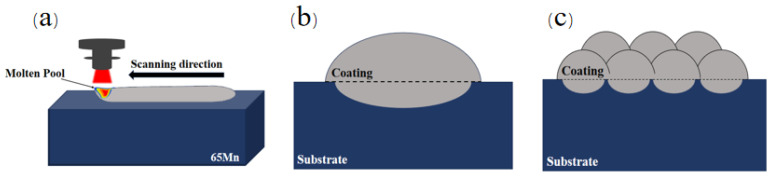
(**a**) Schematic diagram of the laser cladding experiment; (**b**) single-layer laser cladding; (**c**) multi-layer cladding.

Fe901 alloy powder was stirred with ball mill (FP400 model, manufactured by Fritsch GmbH, Idar-Oberstein, Germany) for 3~5 h to mix elements uniformly. It was then dried in a desiccator for 4 h to obtain the Fe901 alloy powder required for the experiment. The elemental composition of the powder is shown in Table 2. The powder particles are spherical in shape, with small particle size and good flowability and are easy to deliver. The coating was prepared with a laser cladding machine system—HLC30 dual cylindrical powder feeding laser (Dazu Laser, Shenzhen, China). The experimental principle is shown in Figure 1. The laser cladding process parameters are given in Table 3.

### 2.2. Methods of Analysis

The required metallographic test pieces are machined from the samples by wire-cutting machine, mounted by metallographic tester, and then the surfaces of the mounted pieces are ground and polished until the surfaces are bright and free from scratches. The coating was etched with metallographic etching solution for 30 s, and the residual etching solution was washed away with alcohol and dried. To ensure reproducibility, all tests, including microstructure observations and measurements, were conducted in triplicates. The coating’s microstructure was observed through scanning electron microscopy (SEM), while the content and distribution profiles of the samples were measured using an energy spectrometer. The composition of the coating phase was characterized using an X-ray polycrystalline diffractometer (model X PERT3 POWER, Panaco, Malvern Panalytical, Malvern, UK). The microhardness of the material was measured using an HV-1000 microhardness tester (Sinowon Innovation Metrology Manufacture Limited, Dongguan, China). The test load traveled a distance of 200 μm, the loading volume load was 25 N, and the wear time was 50 s. The microhardness tests were repeated three times to ensure consistency and accuracy. The friction coefficients of Fe901 fusion cladding and substrate 65 Mn were measured by RTECMFT-3000 (Rtec Instruments, San Jose, CA, USA) friction tester under the conditions of 50 N force, 2 Hz frequency, 10 mm reciprocating stroke, and 1800 s wear time, as per ASTM G99-17 standard [23]. These wear tests were also conducted in triplicate to verify the results. Wear traces of the coating were imaged by scanning electron microscopy to meticulously analyze the wear mechanism. The grinding ball of the friction vice is selected as GCr15 bearing steel ball with a 4 mm diameter and 58-62 HRC hardness. Wear volumes and surface profiles were analyzed and measured using a three-dimensional profilometer (model NDI Vicra SCAN, NDI, Waterloo, ON, Canada), with all measurements repeated three times for reproducibility.

## 3. Results and Discussion

### 3.1. Physical Phase Analysis of Fe-Based Alloy Coatings

**Figure 2:**
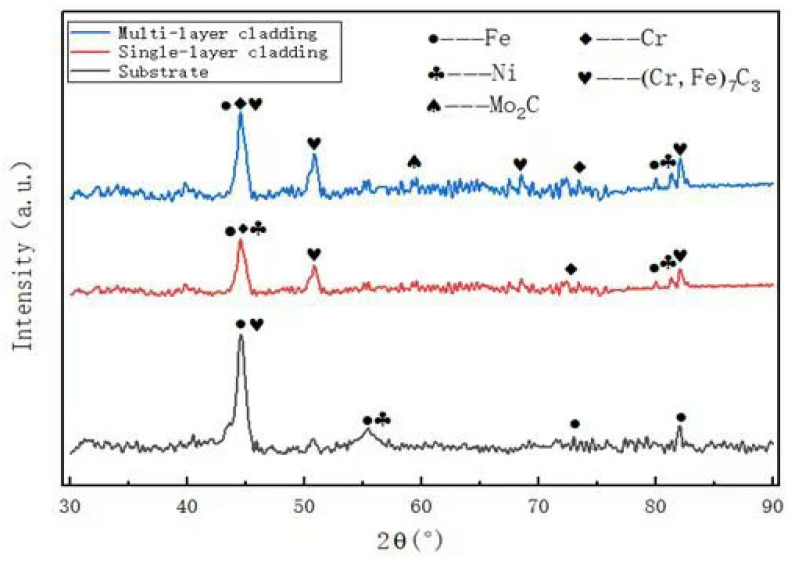
XRD pattern of coating and substrate. The identified phases include α-Fe (PDF#06-0696), Cr_7_C_3_ (PDF#36-1482), Mo_2_C (PDF#35-0787), Ni (PDF#04-0850), and Cr (PDF#06-0694).

The XRD patterns of the laser melted Fe901 alloy coating and the substrate are presented in Figure 2. The coating consists of σ-phases, solid solutions, and carbides (Cr_7_C_3_ and Mo_2_C), as well as small amounts of other compounds. Metal compounds such as Co_7_Mo_6_ and CoMo_2_Ni make up the σ phase. Lattice boundaries change during the formation of solid solutions and various compounds, stimulating the properties of each element, leading to strengthening or weakening of interactions. The ductility and plasticity of the coating are affected by the σphase content due to its brittle nature. Excessivσ-phase can increase crack sensitivity and affect overall quity. While σ-phase can enhance coating hardness, it should be used judiciously to avoid these negative effects. Depending on the material properties of Ni, more austenite can be produced during the cladding process to prevent the formation of excessive σ-phase, absorb residual stresses and reduce cracking. Molybdenum has a relatively low enthalpy and readily forms compounds with other elements. Additionally, pure iron is transformed from one structure to another as the temperature increases; however, Mo is able to induce the transformation of the body-centered cubic lattice (α-Fe) to produce the corresponding solid solution to play a reinforcing role. During rapid cooling, some of the chromium and molybdenum are precipitated at grain boundaries, forming compounds such as Cr_7_C_3_ and Mo_2_C. The strength of the 65 Mn base steel is enhanced by the superior wear resistance and hardness of the carbides in the coating.

### 3.2. Microstructure Analysis

**Figure 3:**
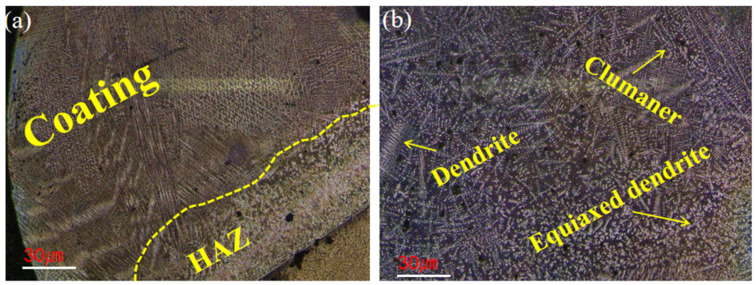
The OM images of the laser-cladded Fe901 coating: (**a**) three typical regions of laser cladding, including the cladding zone, heat-affected zone, and substrate; (**b**) microstructure after corrosion.

The optical microscope (OM) clearly shows the cladding area and the HAZ when observing the microstructure of the corroded coating. Bright planar crystal bands appear in the region where the HAZ meets the coating, as shown in Figure 3. Due to the temperature gradient in the HAZ and the relatively large solidification rate, heat transfer intensity during laser processing is higher from the top downward, while the microstructure grows from the bottom upward. As a result, the heat-affected zone of the microstructure grows slowly and forms planar crystals. This is caused by transient liquid phase diffusion between the Fe901 alloy coating and the base 65 Mn steel, indicating that the melt-coated layer has excellent molding quality, with no defects such as porosity, microcracks, etc. The dilution rate of the coating is low, and produces a very successful metallurgical bond between the substrate and the coating.

**Figure 4:**
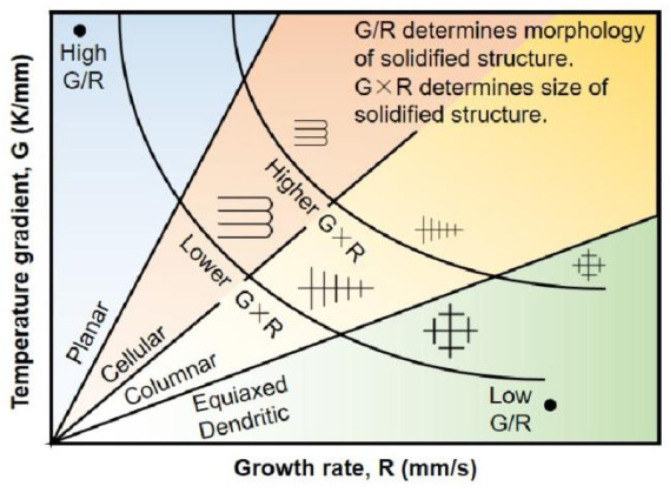
Grain changes during laser cladding pool solidification.

**Figure 5:**
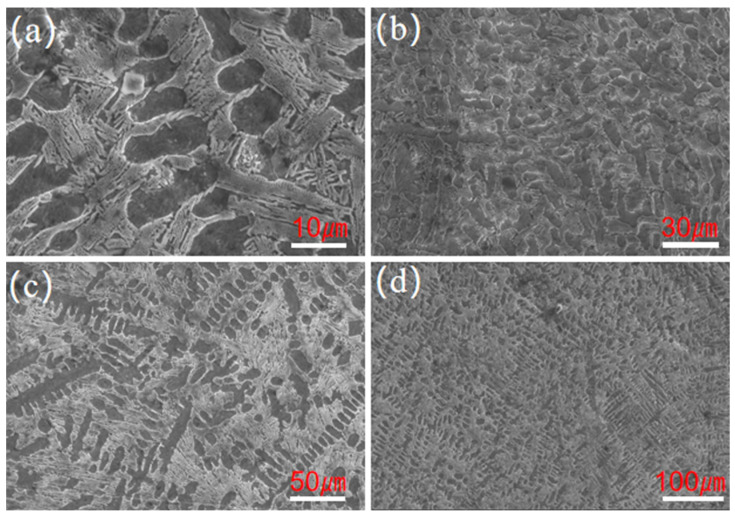
SEM images of the laser-melted Fe901 alloy cladding: (**a**) localized microstructure of the lower layer; (**b**,**c**) microstructure of the intermediate layer; (**d**) microstructure of the upper layer.

Figure 4 shows the grain changes during solidification of the laser-melted molten pool and Figure 5 displays the SEM image showing the coating of the laser fused Fe901 alloy. A white reticulation with uniform appearance was observed. The relationship between two parameters, temperature gradient (G) and solidification rate (R), determines the evolution of solid–liquid interface organization during laser cladding. This is because the process involves rapid heating and cooling, which leads to solidification. Figure 4 shows how crystal shape is affected by temperature gradient and solidification rate. According to this principle, high G/R and low G × R planar crystals form in the lower part of the melt pool. Instead, finer equiaxed crystals are generated in the upper part of the melt pool, as shown in Figure 5b. When the intermediate coating begins to solidify, the metal powder melts at high temperatures under the action of the laser on the substrate at lower temperatures, at which time the temperature gradient G reaches a maximum, and the solidification rate R is close to a minimum, resulting in the formation of the crystal organization on the surface of the substrate is a planar growth of extension and expansion. which is in agreement with the study of Luo et al. [24]. As the cladding process continues, the temperature gradient (G) decreases, and the alloy coating at the bottom of the molten pool solidifies rapidly. Consequently, the solidification rate (R) gradually increases, causing the G/R ratio to decrease. As a result, the crystalline organization is altered, leading to the production of a microstructure in which dendritic and polygonal grains are co-distributed. With an increasing number of fused cladding layers and increasing height of fused cladding, the G/R value continues to decrease during solidification and the organization changes to a sub-eutectic organization, as shown in Figure 5c. The G/R ratio reaches its minimum in the final stage of fusion cladding. It favors the formation of new crystalline compounds, reduces the production of a large number of columnar strong dendrites, and increases the finer, more uniform and compact isometric crystal structure, similar to the results of the study by Xu et al. [25]. Furthermore, the surface of the intermediate layer is remelted and resolidified when a new fused layer is prepared on the surface of an intermediate layer [26]. This process leads to the creation of some of the small isometric crystals, as depicted in Figure 5d.

**Figure 6:**
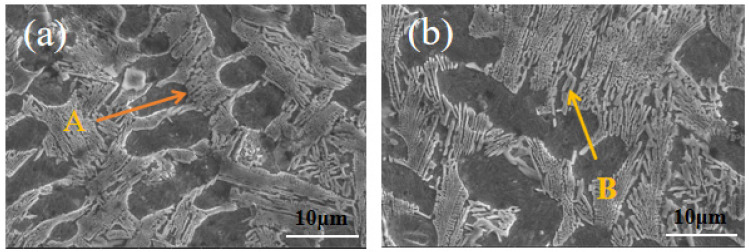
SEM images of laser cladding Fe901 alloy coating spot scanning: (**a**) single-layer laser cladding test image; (**b**) multi-layer laser cladding test image.

**Figure 7:**
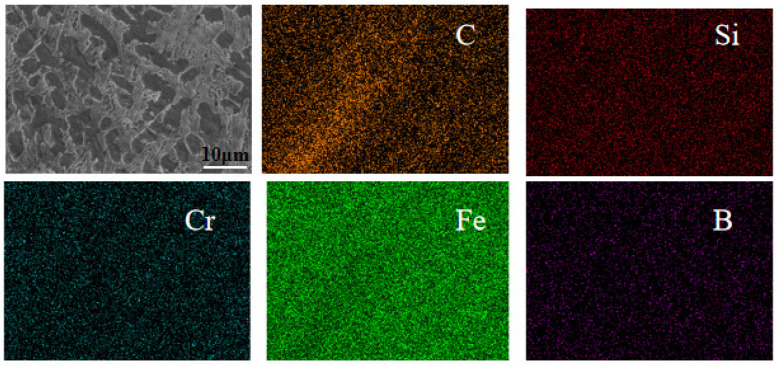
EDS image of SEM face scan of laser cladding Fe901 alloy coating.

Figure 6 shows the SEM image of the laser-melted coating of the Fe901 alloy that was spot-swept. Figure 6a illustrates a single-layer laser-cladded coating, composed mainly of dendritic structure, while Figure 6b depicts a multi-layer laser-cladded coating consisting of eutectic structure between dendrites. Test point A is located in the dendritic organization and test point B is located in the interdendritic eutectic organization. Table 4 shows the EDS elemental distribution, indicating that both dendritic and interdendritic structures contain elements B, Fe, Cr, C, Si, and Ni. The dendritic structure has a higher Fe content than the interdendritic structure, while the interdendritic structure has a higher Cr content than the dendritic structure. In addition, interdendritic structures have higher C element content than dendritic structures, while interdendritic structures have higher Ni element content than dendritic structures. Figure 7 shows that the element Fe is widely distributed throughout the tissue without significant segregation and is more abundant in the dendrites. In contrast, elements such as Cr and B are more densely distributed among the dendrites, and segregation occurs at grain boundaries. Due to their similar atomic radii, Cr and Fe can easily form an α-(Fe,Cr) solid solution, which provides solid solution strengthening. Elements such as Cr, B, C, and Ni tend to segregate at grain boundaries, easily forming network-like and layered structures of hard phases and eutectic phases with Fe elements between dendrites. These structures are interwoven and widely distributed in the coating, playing a role in skeleton reinforcement. The face scan did not detect the presence of element Ni, which may be present in low levels. Therefore, it can be concluded that the dendritic organization mainly contains body-centered cubic lattice (α-Fe) and solid solution (α-(Fe,Cr)). Meanwhile, interdendritic organization mainly disperses hard phases ((Cr,Fe)_7_C_3_), eutectic and strengthening phases, and solid solution grids (α-(Fe,Cr)). The microstructures revealed that the crystal and grain distributions of the multi-layer laser-melted coatings were better than those of the single-layer laser-melted coatings. This enhances the hardness and microstructural properties of the substrate.

### 3.3. Microhardness Analysis

**Figure 8:**
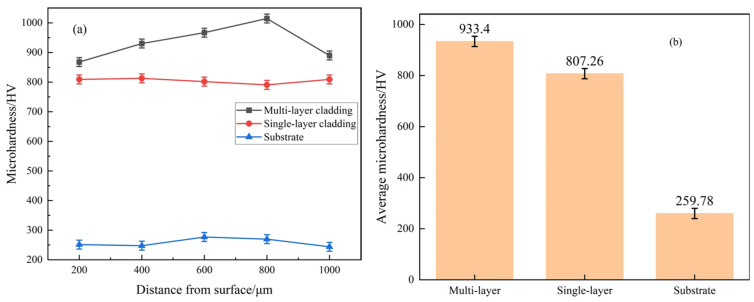
(**a**) Microhardness distribution of Fe901 alloy coatings and substrates; (**b**) average microhardness of Fe901 alloy coatings and substrates.

The microhardness profile and average microhardness variation profile of the Fe-based alloy coating along the depth variation are presented in Figure 8. Figure 8a shows that the microhardness of the multi-layer laser-melted coating is the largest, and the values at each test point are greater than the hardness of the single-layer laser-melted coating. Moreover, the microhardness of the laser-melted multi-layer coating is much greater than the substrate. The microhardness of the multi-layer cladding is the highest, followed by the single-layer cladding, and the substrate is the lowest. It can be seen that the Fe901 laser cladding improves the hardness of the 65 Mn base steel, but the effect is more pronounced for the multi-layer laser cladding. Figure 8b shows that the average hardness of the multi-layer cladding coating, single-layer cladding coating, and substrate are 933.40 HV, 807.26 HV, and 259.78 HV, respectively. The error range is ±20. The microhardness of multilayer lasered coating exceeds that of single-layer lasered coating by a factor of 1.16 and that of substrate by a factor of 3.59. Hardness gradually decreases from the coating surface to the HAZ and then to the substrate. Due to the combined influence of the flow field and temperature gradient, reinforcing phases tend to float toward the top of the melt pool. This behavior reduces their concentration at the bottom of the coating, resulting in localized stress imbalances and potential hardness reduction. Despite this, the microhardness in the cladded region remains relatively uniform, showing no abrupt decrease from the cladding layer to the heat-affected zone (HAZ). However, the rapid solidification of the melt pool may lead to the formation of various defects in the cladding layer, such as inclusions, porosity, and surface irregularities, all of which can degrade the overall mechanical integrity of the coating. It is important to note that this statement is objective and avoids any subjective evaluations. In the HAZ, the decline in hardness is abrupt and much more rapid. The decrease in strength is caused by two factors: the reduction in the reinforcing phase and the remelting crystallization of the lower hardness substrate with the cladding material. The analysis above indicates that the Fe901 alloy multilayer laser cladding coating has a substantially higher total hardness than the original hardness of the 65 Mn base steel, thus increasing the industrial production utilization. The SEM images reveal that the coating surface and matrix have distinct microstructures. The coating microstructure consists mostly of fine equiaxed crystals, while the matrix microstructure is tempered sohnite, containing large particles of carburite. The size of the particle morphology was directly reflected by the projection of the nanoparticles on the photographs, namely the transmission electron microscopy method, which measured the average size of the crystal grains of the coatings to be smaller than the average size of the grains of the matrix tissue, which were 5 μm and 10 μm, respectively. According to Hall–Petch theory [27], the hardness of a material is generally negatively correlated with the size of its microstructure’s grain size. This means that as the crystal grain size increases, the hardness decreases. In this experiment, it was found that the hardness of the Fe901 alloy multilayer laser fusion cladding layer was much greater than the hardness of the base 65 Mn steel.

### 3.4. Wear Resistance Analysis

**Figure 9:**
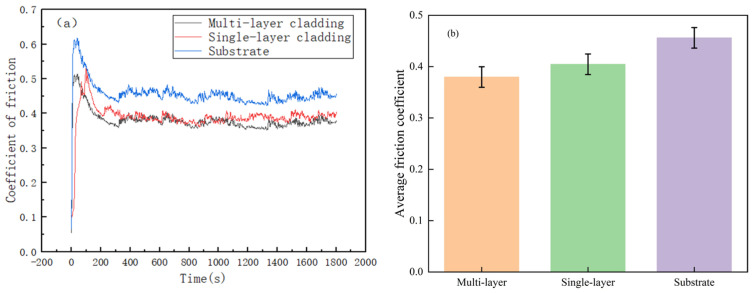
(**a**) Friction coefficient curves of Fe901 alloy coating and substrate; (**b**) average friction coefficient.

Figure 9 displays the coefficient of friction profile of the coating against the substrate. Specifically, Figure 9a illustrates that all friction coefficient curves experience a sharp increase in the initial stage. In the early stages of wear, the limited contact area between the wear pair and the sliding friction block results in high friction forces on the outer layer of the fusion cladding and high contact stresses on the substrate surface, resulting in severe wear and highly variable coefficients of friction. It is important to note that this phenomenon occurs due to the small contact area during the initial stage of wear. During wear experiments, the coating gradually wears down, causing a decrease in the surface hardness and accelerating wear. The use of an abrasive pair alters the morphology of both the fused cladding and the substrate. Additionally, the abrasion process modifies the surface roughness of both materials, leading to an increase in contact area and a reduction in abrasion rate until a steady state is achieved. As depicted in Figure 9b, the average friction coefficients of the multilayer laser-melted coating, the single-layer laser-melted coating, and the substrate are 0.38, 0.41, and 0.46, respectively. The friction coefficients of the cladding layers are lower than those of the substrate. Additionally, the single-layer laser-melted coatings have lower friction coefficients than the multi-layer laser-melted coatings. In summary, both the multi-layer laser cladding coating and the single-layer laser cladding coating demonstrate higher wear resistance than the substrate. However, the Fe901 coating produced by multi-layer laser cladding exhibits superior wear resistance. The wear resistance of the cladding layer is positively correlated with its hardness [28,29,30]. Multi-layer laser cladding coatings contain more carbides and other hard phases that increase the hardness of the cladding layer. The higher hardness increases the resistance of the material surface to fracture and can effectively inhibit wear of the wear surrogates, thereby reducing the coefficient of friction of the cladding layer.

**Figure 10:**
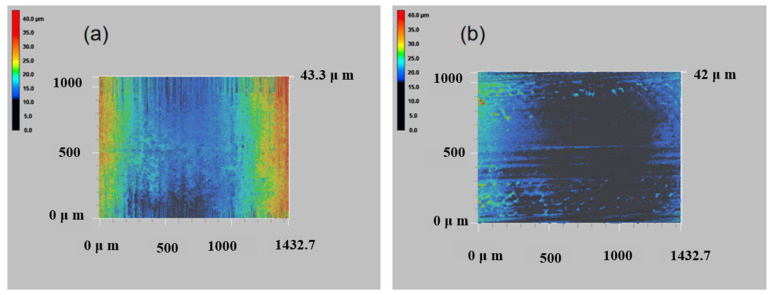
Three-dimensional morphology of coated wear surfaces: (**a**) multi-layer cladding; (**b**) single-layer cladding.

**Figure 11:**
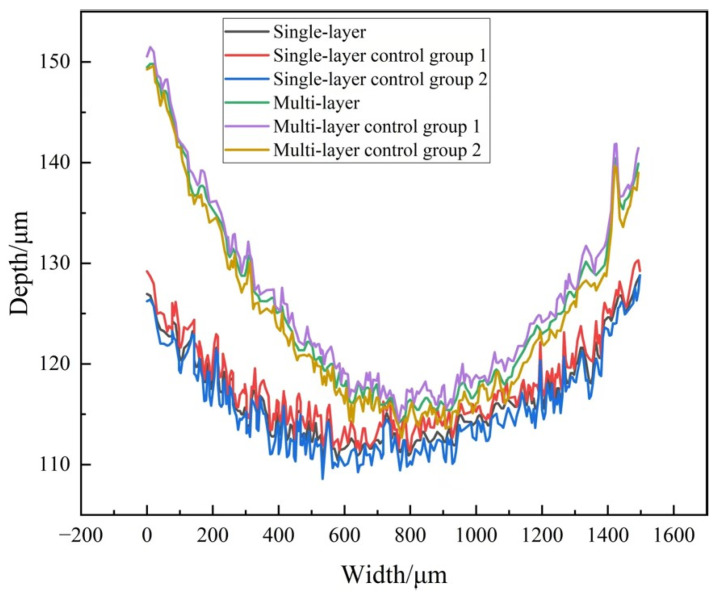
Coating wear profile curve.

Figure 10 displays the 3D wear morphology of the coating. The data shows that the abrasion depth of multi-layer laser-melting coatings is lower than that of single-layer laser-melting coatings when subjected to the same load. The failure of the fused cladding surface is due to abrasive wear and cutting groove wear. During the abrasion process, wear particles are generated between the abrasive member and the abraded part. These particles repeatedly roll or slide on the abraded specimen. The movement of different ways and the transformation of force roles subject the friction vice to friction, causing hard particles to extrude and grind against the surface. This dual role results in cutting, fractures, and other damage to the coating [31]. Under compressive stress, the material’s surface undergoes plastic deformation, resulting in a crater. Friction causes the abrasive material to generate heat, which softens the coating, reduces its hardness, and destroys the surface microstructure. This results in skid marks and flaking. Groove scratches are produced on the surface of the material when the frictional force exceeds its strength limit [32]. Under repeated impact and extrusion of abrasive material, grooves are formed, leading to localized stress concentration and plastic deformation. Simultaneously, energy undergoes a transformation from mechanical to thermal energy, resulting in an interaction within the material. This can lead to fatigue wear and peeling of the material surface, as shown in Figure 11. Therefore, it is evident that multi-layer laser cladding coating is less susceptible to material deformation and wear compared to single-layer fusion cladding. The wear surface of the former only exhibits minimal drilling pits and adhesion off. These results demonstrate that the strength of the multi-channel fusion cladding layer is high, which effectively slows down wear.

**Figure 12:**
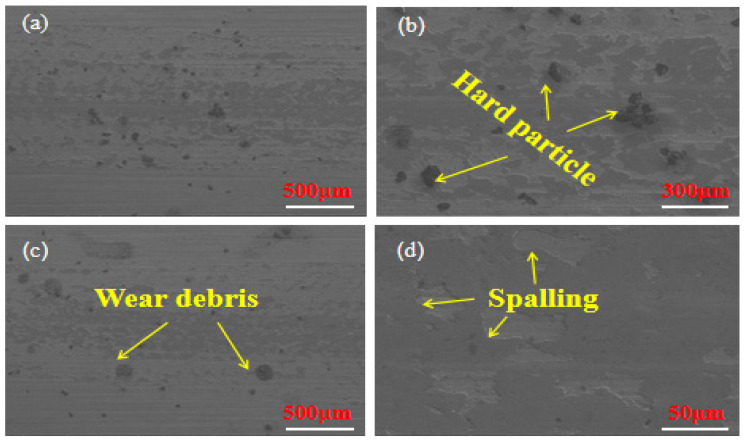
Wear surface SEM of multi-layer fused cladding layers: (**a**) localized wear morphology of wear traces of multi-layer fused cladding layers; (**b**) wear morphology of hard particles; (**c**) wear morphology of debris; (**d**) wear morphology of spalling.

Figure 12 shows the SEM of the wear surface of the multilayer laser fusion coating. The wear mechanism of the multi-layer laser cladding coating is fatigue wear and abrasive wear, as shown in Figure 10a; the worn surface has almost no plastic grooves or pits. Figure 10b shows numerous hard particles present on the surface of the multi-layer laser cladding coating. The presence of large amounts of carbide ((Cr, Fe)_7_C_3_) hard phases increases the wear resistance of the coating, which eliminate grooves in the wear surface. As the abrasive particles pass through the carbides, more carbides are added, further enhancing the coating’s wear resistance [33]. The reason for this is that (Cr, Fe)_7_C_3_ can hinder abrasive particles from penetrating the material surface. As a result, the surface only sustains narrow and shallow scratches and grooves, and there is no accumulation of wear debris at the edges of the wear scars. As depicted in Figure 12c, wear spalling debris is a result of continuous friction wear process. The abrasive material is pressed into the friction surface under the action of stress, producing an indentation. This, in turn, causes the surface of the coating to shed layers or scales of spalling debris. Figure 12d shows the friction surface that is subjected to cyclic contact stresses generated by the abrasive. This causes the coating to flake off due to fatigue. The stress caused by abrasive particles can lead to the formation of microcracks, which propagate along the boundaries of carbides. Nanoscale debris is present on both sides of the fatigue crack as a result of repeated surface friction. Under the action of abrasion, the metal on the surface of the coating flakes off. In actual wear, the wear of the coating is affected differently by multiple factors such as the geometrical properties of the abrasive, the relative hardness, the sliding speed, the magnitude of the load, and the number of repetitions of friction. Furthermore, research has shown that (Cr,Fe)_7_C_3_ impedes dislocation motion and resists plastic flow, resulting in improved wear resistance [34].

## 4. Conclusions

Fe901 multi-layer laser fusion coating on 65 Mn steel substrate produced by laser fusion coating technology. The physical phase and microstructure of the coatings were analyzed, and the microhardness and friction properties of the coatings were investigated. The preparation method of multilayer laser-melted iron-based coatings proposed in this paper is useful for research and the development of new iron-based coatings with higher hardness and superior wear resistance to provide reference. The study’s main conclusions are summarized below.

1. Multi-layer and single-layer laser cladding coatings were successfully prepared on the 65 Mn substrate. The coatings exhibited a dense structure with no visible pores or cracks on the surface, indicating excellent metallurgical bonding. The Fe901 coating had a uniform composition distribution, consisting mainly of α-Fe and (Cr,Fe)_7_C_3_ phases. Microstructure of multi-layer laser cladding coating was primarily composed of fine equiaxed grains, while the single-layer laser cladding coating was mainly dendritic.
2. The hardness of the multilayer laser cladding is 3.59 times higher than the hardness of the substrate, which is higher than the single-layer laser cladding. This can be attributed to the presence of more (Cr,Fe)_7_C_3_ throughout the multilayer laser clad coating, which inhibits dislocation movement in the lattice and enhances its resistance to plastic deformation.
3. Multi-layer cladding wear resistance is better than single-layer cladding and substrate. Moreover, the mean friction coefficient of the laser clad is much lower than the mean friction coefficient of the substrate. This is due to the positive correlation between the wear resistance and coating hardness. The increased hardness of the multi-layer laser cladding coating results in a reduction in its wear. In terms of wear mechanism, multi-layer laser cladding coatings are mainly abrasive wear and the substrate is adhesive wear. Multi-layer laser cladding produces tight bond strength and forms an excellent wear-resistant skeleton, improving wear resistance.

This research holds significant practical importance for agricultural machinery, as it can enhance the performance and service life of soil-contacting parts, which are subject to harsh working conditions. The findings provide important an reference for improving the wear resistance and durability of agricultural equipment, thus contributing to the advancement of agricultural mechanization.

## Figures and Tables

**Table 1:** Composition of substrate 65 Mn steel (wt.%).

C	Si	Mn	Ni	Cr	Fe
0.60–0.72	0.15–0.33	0.93–1.20	≤0.29	≤0.16	Bal.

**Table 2:** Chemical composition of Fe901 alloy powder (wt.%).

C	Cr	Si	B	Mo	Ni	Fe
0.15	13.5	1.3	1.6	0.8	1.8	Bal.

**Table 3:** Laser cladding process parameters.

Content	Numerical
Output power (W)	1200
Processing rate (mm/min)	800
Spot size (mm)	5
Overlap rate (%)	50

**Table 4:** Distribution of EDS elements.

Test Points	B	Fe	Cr	C	Si	Ni
Point A	1.06	80.32	12.43	1.16	1.44	0.73
Point B	1.13	71.49	18.56	1.98	1.49	1.05

## Data Availability

The original contributions presented in this study are included in the article. Further inquiries can be directed to the corresponding author.
